# Synthesis, Molecular Docking, and Antimalarial Activity of Hybrid 4-Aminoquinoline-pyrano[2,3-c]pyrazole Derivatives

**DOI:** 10.3390/ph14111174

**Published:** 2021-11-17

**Authors:** Mohd Asyraf Shamsuddin, Amatul Hamizah Ali, Nur Hanis Zakaria, Mohd Fazli Mohammat, Ahmad Sazali Hamzah, Zurina Shaameri, Kok Wai Lam, Wun Fui Mark-Lee, Hani Kartini Agustar, Mohd Ridzuan Mohd Abd Razak, Jalifah Latip, Nurul Izzaty Hassan

**Affiliations:** 1 Department of Chemical Sciences, Faculty of Science and Technology, Universiti Kebangsaan Malaysia (UKM), Bangi 43600, Selangor, Malaysia; asyrafsham@yahoo.com.my (M.A.S.); amatulhamizahali@yahoo.com.my (A.H.A.); nhz2nurhaniszakaria@gmail.com (N.H.Z.); jalifah@ukm.edu.my (J.L.); 2 Institute of Science, Universiti Teknologi MARA, Shah Alam 40450, Selangor, Malaysia; mohdfazli@uitm.edu.my (M.F.M.); asazali@uitm.edu.my (A.S.H.); zurina@uitm.edu.my (Z.S.); 3 Drugs and Herbal Research Centre, Faculty of Pharmacy, Universiti Kebangsaan Malaysia, Jalan Raja Muda Abdul Aziz, Kuala Lumpur 50300, Malaysia; david_lam@ukm.edu.my; 4 Department of Chemistry, Faculty of Science, Universiti Teknologi Malaysia (UTM), Johor Bahru 81310, Johor, Malaysia; mleewf@gmail.com; 5 Department of Earth Science and Environment, Faculty of Science and Technology, Universiti Kebangsaan Malaysia (UKM), Bangi 43600, Selangor, Malaysia; 6 Herbal Medicine Research Centre, Institute for Medical Research, National Institutes of Health (NIH), Ministry of Health Malaysia, Shah Alam 40170, Selangor, Malaysia; ridzuan.ar@moh.gov.my

**Keywords:** 4-aminoquinoline, antimalarial, docking, hybrid, pyrano[2,3-c]pyrazole

## Abstract

Widespread resistance of *Plasmodium falciparum* to current artemisinin-based combination therapies necessitate the discovery of new medicines. Pharmacophoric hybridization has become an alternative for drug resistance that lowers the risk of drug–drug adverse interactions. In this study, we synthesized a new series of hybrids by covalently linking the scaffolds of pyrano[2,3-c]pyrazole with 4-aminoquinoline via an ethyl linker. All synthesized hybrid molecules were evaluated through in vitro screenings against chloroquine-resistant (K1) and -sensitive (3D7) *P. falciparum* strains, respectively. Data from in vitro assessments showed that hybrid **4b** displayed significant antiplasmodial activities against the 3D7 strain (EC_50_ = 0.0130 ± 0.0002 μM) and the K1 strain (EC_50_ = 0.02 ± 0.01 μM), with low cytotoxic effect against Vero mammalian cells. The high selectivity index value on the 3D7 strain (SI > 1000) and the K1 strain (SI > 800) and the low resistance index value from compound **4b** suggested that the pharmacological effects of this compound were due to selective inhibition on the 3D7 and K1 strains. Molecular docking analysis also showed that **4b** recorded the highest binding energy on *P. falciparum* lactate dehydrogenase. Thus, *P. falciparum* lactate dehydrogenase is considered a potential molecular target for the synthesized compound.

## 1. Introduction

Malaysia has made significant progress in the control of malaria. However, the fatality rate fluctuated throughout the period 2001–2012. The National Strategic Plan for Malaria Elimination 2011–2020 was initiated to stop locally-acquired malaria in Peninsular Malaysia by 2015 and East Malaysia by 2020. To date, reports on the development of resistance towards artemisinin in Southeast Asian countries have initiated a ‘Global Plan for Artemisinin Resistance Containment’ by the World Health Organization (WHO) Global Malaria Program in 2011 to prevent the emergence and spread of artemisinin resistance. According to the public health perspective, the goal of treatment of malaria is to reduce transmission of the infection to others by reducing the infectious population and the spread of resistance to antimalarial medicines. The development of a multi-therapeutic approach, advanced analogs, and identification of novel leads were examined as potential alternatives because artemisinin-based combination therapy (ACT) is the only standard treatment recommended by the WHO facing similar situations of antimalarial resistance [1]. Different classes of drugs have been used to combat malaria, such as quinoline-based drugs (e.g., quinine, chloroquine, amodiaquine, primaquine, and mefloquine), artemisinin and peroxide-based drugs (e.g., artemisinin, artemether, artesunate, and dihydroartemisinin), folate synthesis inhibitor (e.g., proguanil, cycloguanil, dapsone, sulfadoxine, and sulfalene), antibiotics (e.g., doxycycline and clindamycin) and various antimalarial drugs (e.g., atovaquone, halofantrine, and deferoxamine). Thus, there is an urgent need to develop new, promising, and economic antimalarial agents [2]. A hybrid drug concept can be employed to covalently link two or more active pharmacophores, which may act on multiple targets in addressing the drug resistance issue.

Morphy et al. [3] and Meunier et al. [4] have introduced the concept of hybrid molecules and hypothesized that it might overcome the re-emergence of drug resistance. The idea of generating hybrid molecules, such as conjugates in which two or more pharmacophoric chemical entities were linked (stable or metabolized to provide free pharmacophores inside the human body), was believed to act simultaneously on two different conventional targets. The main advantages of the combination therapy strategies are reduced risk of drug–drug interactions, patient compliance, and predictable pharmacokinetics [5]. A fixed-dose combination relied on the different levels of bloodstream uptake due to changes in solubility of the partner drug. However, the fine-tuning required to ensure similar blood levels of drugs administered in the same tablet can be surpassed with a hybrid drug. The uptake capacity can contribute to the bioavailability if one scaffold of the hybrid molecule is more soluble. Besides, the linker employed in chloroquine-pyrimethamine hybrids has two ethylene oxide units, which hydrogen bonds with the water molecules. It is most likely to contribute to its excellent solubility in acidic and neutral media [6].

Joubert et al. [7] showed that drug-likeliness characteristics in artemisinin-acridine hybrids have extremely low solubility and absorption levels under physiological conditions and undefined blood-brain barrier penetration levels. They later proposed that blocking polar primary/secondary amine groups in the intermediates reduces hydrogen bond formation. Thus, new metabolic liabilities may arise by molecular hybridization, leading to loss or gain of good absorption, distribution, metabolism, excretion, toxicity, and pharmacokinetics (ADMET/PK) properties of the individual pharmacophoric entities [8].

The 4-aminoquinoline pharmacophore is still indispensable for combating malaria. It is the most sought-after antimalarial agent for chemical modification due to its ease of synthesis, low cost, and ease of use [9]. The SAR studies on 4-aminoquinolines [10] propose that 7-chloro and 4-amino groups are critical for the inhibitory effect of hemozoin formation and help the drug accumulate in the parasite’s acidic food vacuole [11]. Substitution of 7-chloro by other groups such as Br, OCH_3_, CH_3_, or CF_3_ leads to decreased activity. In the same way, the substitution of 4-amino groups by S or O groups will reduce antimalarial activity. The nitrogen of 4-aminopyridine and the tertiary amine at the side chain are essential for activity as they help the drug accumulate in the parasite cell by becoming protonated. The optimal carbon chain length was between 2 and 3 to help the compound retain the antimalarial activity against the chloroquine resistance of *P. falciparum* [12].

4-Aminoquinoline-based isatin derivatives have been designed based on a multi-therapeutic strategy. Isatin derivatives were functionalized with the thiosemicarbazone moiety that could inhibit *P. falciparum*-derived cysteine proteases [13]. These proteins are essential for the degradation of hemoglobin during erythrocytic parasite development. Thus, the 4-aminoquinoline scaffold could promote accumulation and heme-binding, whereas the isatin group inhibits *P. falciparum* cysteine proteases. The length and nature of the linker may exert a profound influence on the antimalarial effect of the conjugates. In this study, the 4-amino groups will be linked with the ethyl chain (two carbon atoms) that may increase activity against chloroquine-resistant strains. Hybrids with a linker chain length greater than three carbon atoms were less potent than chloroquine [8]. Besides conjugating 4-aminoquinolines with established antiplasmodial targets such as cyclic peroxides, newer antiplasmodial agents based on various scaffold, such as imipramine and pyrimidine and isatin derivatives, were introduced to help with the drug resistance. Most of the published studies on the design and synthesis of antimalarial hybrids involved different pharmacophore components such as endoperoxide–quinoline-based hybrids, endoperoxide–chalcone-based hybrids, and trioxaquine–quinoline based hybrids [7,8].

**Figure 1:**
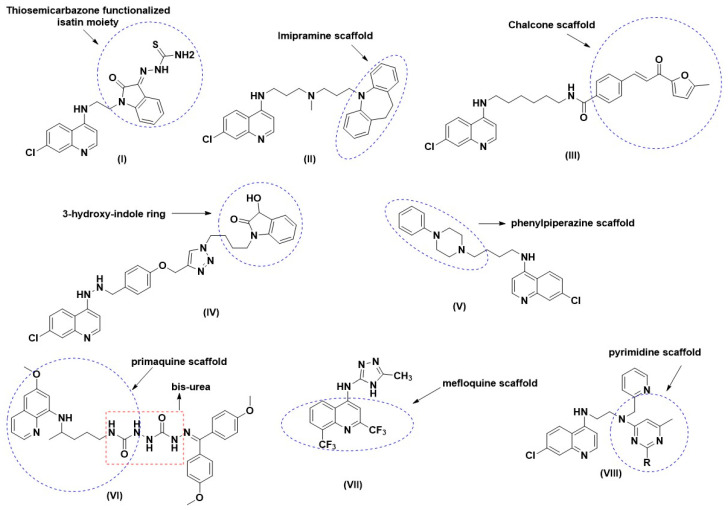
Miscellaneous molecular hybrid based on 4-aminoquinolines, primaquine, and mefloquine (**I**–**VIII**) reported from 2005–2019.

A new scaffold of thiosemicarbazone-functionalized isatin (I) was incorporated onto the 4-aminoquinoline scaffold to inhibit the falcipain-2-enzyme, an essential cysteine protease in *P. falciparum* responsible for the degradation of hemoglobin [13]. The hybrids demonstrated good antimalarial activity against chloroquine-sensitive strains, chloroquine-resistant strains of *P. falciparum*, and the recombinant falcipain-2 enzyme [13]. Burgess et al. [14] linked 4-aminoquinoline with imipramine (II), which exhibited promising in vitro and in vivo efficacy against tested *P. falciparum* strains. Smit and N’Da [15] reported novel 4-aminoquinolinyl-chalcone amides (III) having IC_50_ values in the range of 0.04–0.5 μM against the 3D7 strain (chloroquine-sensitive) (Figure 1).

Kumar et al. prepared a new series of phenolic ether-based 1*H*-1,2,3-triazole tethered 3-hydroxy-indole-7-chloroquine hybrids (IV). Another class of 4-aminoquinolines linked with phenylpiperazines (V) showed superior activity against the K1 strain as compared to chloroquine [16]. Primaquine hybrids containing a bis-ureas scaffold developed by Pavic et al. [17] showed potential antimalarial activity against the liver stage of *P. berghei*, with 3-(4-chlorophenyl)-1-[({4-[(6-methyoxyquinolin-8-yl)amino]pentyl}carbomyl)amino] urea (VI) being the most active compound (IC_50_ = 42 nM; cytotoxicity/activity ratio > 2000). *N*-substituted aminoquinoline-pyrimidine hybrids (VII) synthesized by Maurya et al. [18] were found to have potent in vitro antimalarial activity against both chloroquine-sensitive D6 and chloroquine-resistant W2 strains of *P. falciparum*. Furthermore, they suggested that heme may be the probable target of these hybrid molecules. Later in the same year, da Silva et al. [19] tested *N*-(5-methyl-4*H*-1,2,4-triazol-3-yl)-2,8-bis(trifluoromethyl)quinolin-4-amine (VIII) against *P. berghei*-infected mice. This compound was active on the 5th day after infection, reducing parasitemia by 66%, consistent with its in vitro activity.

An important class of heterocyclic scaffold, pyrazole, was first evaluated for its in vitro antimalarial activity against *P. falciparum* by Satasia et al. [20] based on pyrido [2,3-d] pyrimidinedone derivatives. The compound exhibited good antimalarial activity (*P. falciparum* IC_50_ = 0.033 μg/mL). In the following year, Insuasty et al. prepared pyrazolines and pyrazolo[3,4-b][1,4]diazepines derivatives, and the compound was found to be active against the *Plasmodium* parasite (IC_50_ = 11.3 ± 2.3 μg/mL) [21]. Another series of pyrazole derivatives were evaluated by hybridization with five-membered heterocyclic moieties such as thiazoles, thiazolidinones, 1,3,4-thiadiazoles, and pyrazolines and demonstrated in vivo antimalarial activity against *P. berghei* infected mice. Bekhit et al. further examined pyrazole derivatives in vitro antimalarial activity against chloroquine-resistant (RKL9) strains of *P. falciparum* (IC_50_ = 0.0368 ± 0.008 μM), which is five-fold higher than chloroquine [22]. Subsequently, a fluoro substituted pyrazole nucleus conjugated with 1,3,4-oxadiazole scaffolds showed excellent antiplasmodial activity against *P. falciparum* compared to quinine (0.826 μM) [23].

**Figure 2:**
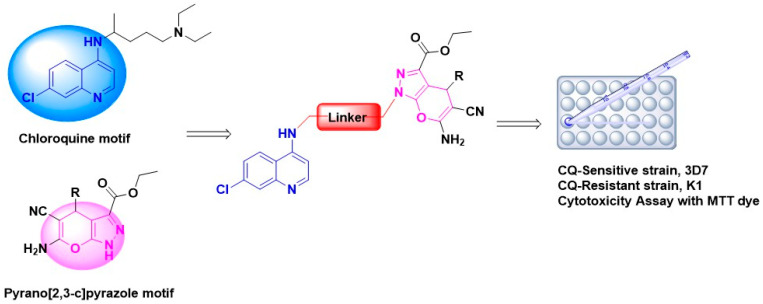
The component of hybrid compounds comprised of chloroquine motif (in blue) and pyrano[2,3-c]pyrazole moiety (in pink).

Nevertheless, to date, no attempt has been made in conjugating 4-aminoquinoline scaffold and pyrano[2,3-c]pyrazole as molecular hybrids for antimalarial agents. In this study, we would like to functionalize the -NH of ethyl linker with pyrano[2,3-c]pyrazole scaffold and study the effect on antimalarial activity. In such a case, functionalization at the -NH of the ethyl linker chain with a heterocyclic group may increase the lipophilicity of the molecule. Introducing this heterocyclic component at this alkyl side chain is vital for antimalarial activity as they help the hybrid drug accumulate in the parasite cell by becoming protonated. The prediction of good bioavailability was carried out using an in silico approach (ADMET/PK profile). Drugs with less than ten rotatable bonds and topological polar surface area less than 140 Å^2^ are more likely to show good bioavailability. All hybrid compounds will be subjected to in vitro antimalarial assays against *P. falciparum* 3D7 and K1 cultures, whereas cytotoxicity assay will be carried out using 3-(4,5-dimethylthiazol-2-yl)-2,5 diphenyl tetrazolium bromide (MTT) assay. In addition, molecular docking analysis on *P. falciparum* lactate dehydrogenase will be conducted to study a potential molecular target of the hybrids (Figure 2).

## 2. Results and Discussion

### 2.1. Chemistry: Reaction and Mechanism

**Figure 3:**
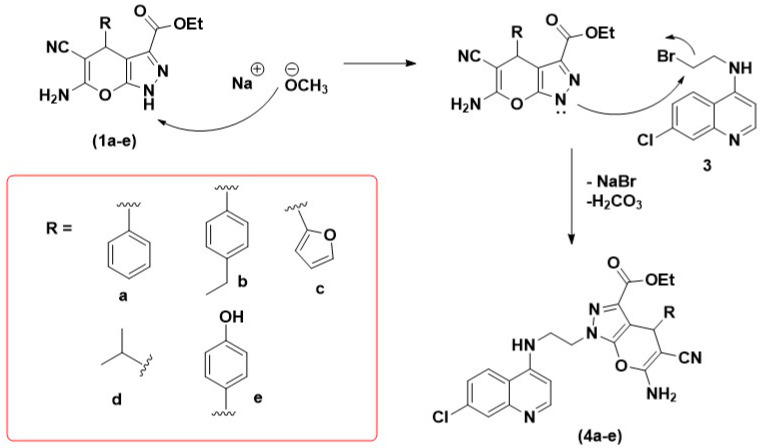
Proposed nucleophilic attack of pyrano[2,3-c]pyrazole derivatives to the 4-aminoquinoline scaffold to obtain the molecular hybrids (**4a**–**e**).

A hybrid scaffold (**4**) was initially synthesized via deprotonation of the acidic proton of the pyranopyrazole (**1**) by the methoxide base. Subsequently, nucleophilic attack of the generated pyrazolium anion to the 4-(bromoethylamino)-7-chloroquinoline (**3**) via S_N_2 reaction furnished 4-aminoquinoline-pyrano[2,3-c]pyrazole hybrid (**4**), as depicted in Figure 3.

**Figure 4:**
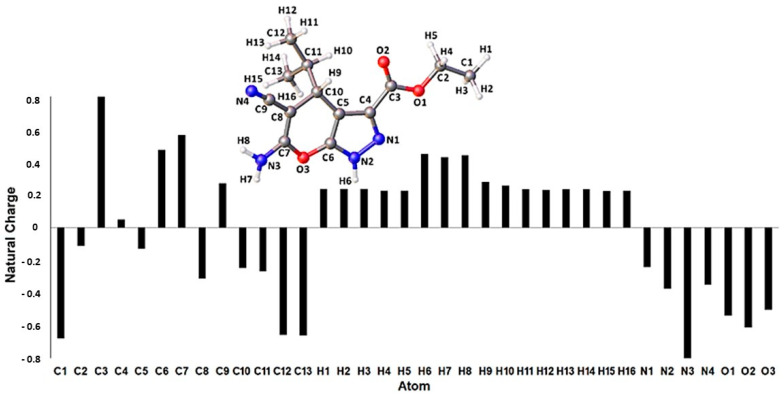
Natural population analysis distribution for the optimized **1d** structure with the atoms labeled.

According to the natural population analysis (NPA), the highest positively charged atom of the pyrano[2,3-c]pyrazole derivative is C3, sandwiched between two electronegative oxygen atoms (Figure 4). The next two highly positive atoms are C6 and C7, which are also located adjacent to heteroatoms of the pyranopyrazole moiety. Subsequently, the three most positively charged hydrogen atoms are associated with the N2 and N3 atoms, i.e., H6 (+0.449) > H8 (+0.441) > H7 (+0.429). This result suggests that H6 is most likely to attract a nucleophile; therefore, it corroborates the anticipated deprotonation of the secondary amine proton [24]. Consequently, the available lone pair of electrons of N2 undergoes a nucleophilic attack on the aminoquinoline to obtain the molecular hybrids **4a**–**e**. Furthermore, the thermodynamic quantity, namely, the standard enthalpy (∆_r_H^θ^) and Gibbs free energy (∆_r_G^θ^) of the reaction, were obtained via DFT calculation. The measured enthalpy change involved synthesizing the hybrids from the deprotonated **1d,** and aminoquinoline was (∆_r_H^θ^ = −555.0 kJ mol^−1^. What is noteworthy is that this exothermic process is also exergonic with the ∆_r_G^θ^ = −518.6 kJ mol^−1^, whereby ∆_r_H^θ^ > ∆_r_G^θ^ and implies that the entropy change, ∆_r_S^θ^ < 0. Therefore, the spontaneity of the reaction depends on the temperature and is spontaneous at lower temperat.

### 2.2. Antimalarial (P. falciparum) and Cytotoxic (Vero Cells) Activities

The antimalarial activities of the series of hybrid molecules were evaluated against chloroquine-sensitive *P. falciparum* 3D7 and chloroquine-resistant *P. falciparum* K1. The results in EC_50_ values (μM) against the 3D7 and K1 strains are presented in Table 1. The thresholds of antimalarial activities were set as follows: EC_50_ < 1 μM as potent, 2–20 μM as active, 21–100 μM as moderately active, 101–200 as weakly active, and EC_50_ > 201 as inactive [25,26]. Data from antimalarial assessment showed that the hybrid molecules **4a**, **4b**, **4c,** and **4e** exhibited potent inhibitory effect against the 3D7 (chloroquine-sensitive) strain with EC_50_ values of 0.19 ± 0.07 μM, 0.0130 ± 0.0002 μM, 0.113 ± 0.002 μM, and 0.026 ± 0.009 μM, respectively. Besides the profound effects against the chloroquine-sensitive strain, the hybrid molecules of **4a**, **4b,** and **4d** also possessed potent inhibition on the chloroquine-resistant strain (K1) with EC_50_ values of 0.25 ± 0.03 μM, 0.02 ± 0.01 μM, and 0.30 ± 0.01 μM, respectively. The antimalarial effects of **4c** and **4d** against K1 and 3D7 were determined to be active, as observed by the EC_50_ values in ranges of 2–20 μM. The EC_50_ value of **4b** against 3D7 was about ten times more than chloroquine, while the EC_50_ value of **4b** against K1 was 100 times more than artemisinin.

The results of the MTT assay against the Vero mammalian cell line revealed that the hybrid molecules **4a**, **4b**, **4c**, and **4d** exhibited low to moderate cytotoxic activities, with CC_50_ ranging from 17 to 103 µM. Based on the cytotoxic threshold from Burger and Fiebig, pure compounds are classified as highly toxic to normal mammalian cells when the IC_50_ value obtained is less than 10 µM or 4 µg/mL [27,28]. To understand the efficacy and potential of the hybrid molecules as safer and selective drugs, we calculated the selectivity indexes from EC_50_ values of antimalarial and CC_50_ values of cytotoxic tests. The calculation of SI values for the inhibition on 3D7 strain revealed that the hybrid molecules (**4a**, **4b**, **4c**, and **4e**) that exhibited potent antimalarial effects have higher SI values that range from 355–1354. The high SI value showed that the antimalarial efficacy of these hybrid molecules is higher than the cytotoxic effect as an indication of its selective inhibitory properties toward the parasite. The calculation of SI values for the inhibition on the K1 strain showed that the hybrid molecules **4a**, **4b**, and **4e** exhibited high SI values (ranges of SI = 287–880). The high SI values demonstrated by these hybrids are parallel to the potent inhibitory effects on the chloroquine-resistant strain. All hybrid molecules have high selective inhibition depending on the strain of parasites. Furthermore, the low resistance index values shown by the hybrid molecules **4a**, **4b**, and **4d** indicate that these molecules’ antimalarial effects against the K1 and 3D7 strains were comparable for both parasite strains.

**4b** has the most potent parasite inhibitory effects from all tested hybrid molecules, showing nanomolar range potency against both sensitive and resistant *P. falciparum* strains with high SI and low RI values. The combination of both 4-aminoquinoline and pyrano[2,3-c]pyrazole has increased the potency of **4b** against the K1 and 3D7 strains. We suggest that both quinolines and pyrazole pharmacophores are responsible for the antimalarial effects shown by **4b**. As of now, this is the first report on the antimalarial properties of the hybrid 4-aminoquinoline-pyrano[2,3-c]pyrazole derivatives against *P. falciparum* 3D7 and KI. Previous studies revealed that hybrid quinoline-pyrimidine showed significant antimalarial activities in the nanomolar range [29]. Smit and N’Da [15] reported novel 4-aminoquinolinyl-chalcone amides with EC_50_ values of 0.04–0.5 μM against chloroquine-sensitive 3D7 strain comparable to the EC_50_ reported in this study. Pyrazole derivatives have also been tested against *P. falciparum,* exerting potent antimalarial activity [20]. Furthermore, Insuasty et al. [21] have evaluated pyrazolines and pyrazolo[3,4-b] [1,4]diazepines derivatives against *Plasmodium*, and the derivatives showed strong antimalarial activities.

### 2.3. Molecular Docking Analysis

**Figure 5:**
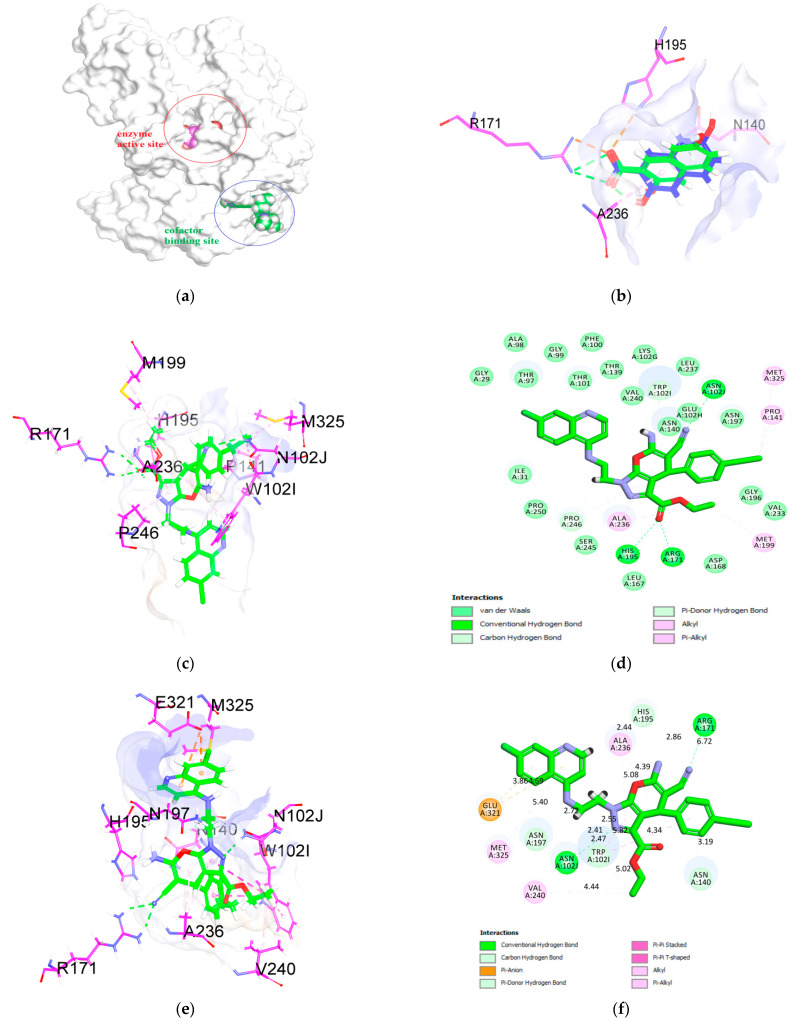
(**a**) Position of the enzyme active site and cofactor binding site of PfLDH. Both the pink and green space-filling calotte models represent the binding position of the co-crystallized inhibitors, 3,5-dihydroxy-2-naphthoic acid and chloroquine. (**b**) Superposition of the docked ligand with the co-crystallized ligand (blue carbon atoms) (RMSD value of 1.32 Å). Best binding pose of compound **4b** in the first (**c**) and second (**e**) cluster results. (**c**) 2D-docking pose of compound **4b** in the first (**d**) and second (**f**) cluster results.

Because *P. falciparum* highly depends on glycolysis for energy production, we hypothesize that hybrid molecules **4a**–**e** exerted the antimalarial effects by inhibiting the activity of *Plasmodium falciparum* lactate dehydrogenase (PfLDH). It is known that there are two crucial ligand binding sites in PfLDH. The first is the enzyme active site, while the second is the cofactor binding site (Figure 5a). To check the docking efficiency, the co-crystallized ligands, 3,5-dihydroxy-2-naphthoic acid and chloroquine were extracted from their respective protein crystal structures (PDB IDs: 1CET and 1U5A) and re-docked back to their putative binding sites. The docking results showed that the RMSD. between the top-ranked pose and the crystal structure was within the acceptable range (<2 Å) (Figure 5b).

The docking results of compounds **4a**–**e** revealed that they prefer to bind to the active site over the cofactor binding site (data not shown here) of PfLDH based on their predicted binding energies and visual inspection. Compound **4b** recorded the highest binding energy out of the five compounds. This finding suggested that PfLDH is a plausible molecular target of the compound. The compound also exerted the highest inhibitory effect on both *P. falciparum* strains (Table 1). In general, two distinct binding conformations of compound **4b** were observed based on the results clustering. The first binding conformation showed that the pyrano[2,3-c]pyrazole ring of compound **4b** positioned itself in the hydrophobic groove in the active site by forming multiple van der Waals contacts with the adjacent residues, as listed in Table 2. One of the interesting observations is the ability of the carboxylate ester group to form hydrogen bonding interactions with the guanidinium side chain of Arg171 and His195. These interactions are pretty similar to the co-crystallized ligand, 3,5-dihydroxy-2-naphthoic acid. It would be interesting to see how the activity would be affected if a free carboxylic group replaces the ester. Furthermore, it is known that Arg171 is highly conserved in the active site. Apart from that, the quinoline ring lies in the hydrophobic groove made up of Gly29-Ile31 and Thr97-Gly99. The nitrogen atom in the quinoline ring forms a hydrogen bond with the hydroxyl side chain of Thr101. Lastly, the cyano group interacts with the carboxamide side-chain of Asn102 via a hydrogen bond (Figure 5c,d).

In the second conformation, the pyrano[2,3-c]pyrazole ring occupies the center of the active site, while the quinoline ring extends out of the binding region into the solvent area. The electronegative cyano group and the nitrogen atom of the pyrazole ring form hydrogen bonds with the Arg171 and Asn102 residues via hydrogen bonding interactions. Not surprisingly, the quinoline ring also participates in ᴨ-alkyl interaction with Ala236. On the other hand, the ethylbenzene ring forms ᴨ-ᴨ stacking with Trp102 (Figure 5e,f). Based on the binding interactions and energies displayed by the docking results, it is likely that compound **4b** prefers the former.

### 2.4. ADMET Profile of Compounds ***4a***–***e***

Table 3 summarizes the ADMET properties for compounds **4a**–**e**. In general, all compounds exhibited a poor level of human intestinal absorption and low aqueous solubility. This could be solved by hydrolyzing the ester group to a carboxylic acid. In terms of protein plasma binding properties capability, all hybrid compounds are predicted to be non-binders. More importantly, the in silico prediction revealed that compounds **4a**–**e** are generally hepatotoxic. In future studies, we will be focusing on removing the toxicophores or replacing them with safer substituents or groups.

### 2.5. Structure-Activity Relationship

SAR studies on the 4-aminoquinoline pharmacophore show that 7-chloro and 4-amino groups are required for π-π interactions and associations with heme to increase the β-hematin inhibitory activity [10]. Similarly, the quinoline moiety is essential and needed to accumulate the compound in the digestive vacuole. This relationship is the reason why the 4-aminoquinoline scaffold is still relevant in malaria drug discovery. Shorter linkers consisting of two carbon chains are optimal to enhance activity against CQ-resistant and CQ-sensitive strains of the parasite. Pyrano[2,3-c]pyrazole core significantly increases the potency of the synthesized compounds.

**Figure 6:**
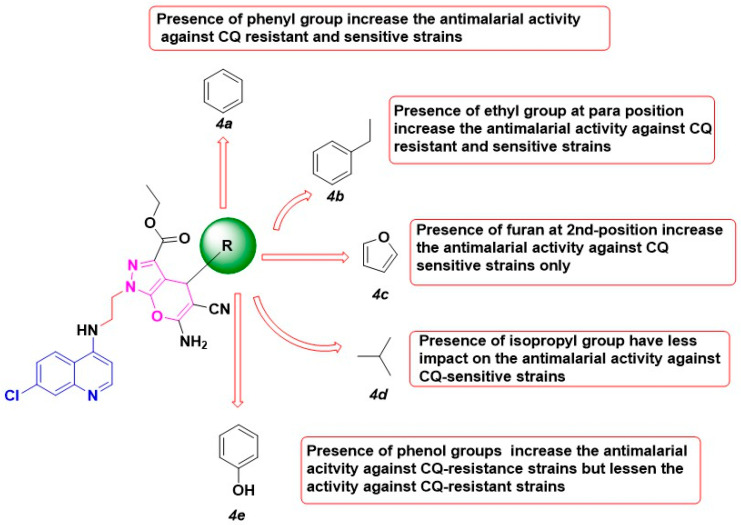
The structure-activity relationship of synthesized compounds, **4a**–**e**.

The presence of bulky and lipophilic groups such as phenyl and furan enhance the antimalarial activity against CQ-sensitive strains 3D7 in **4a**, **4b**, **4c,** and **4e**. However, the removal of the phenyl group in **4d** decreases the potency of the compounds. Contrarily, the furan substitution at the 2nd position generally decreased the activity against CQ-resistant K1 strains. Similarly, the addition of the hydroxyl group in **4e** unfavorably reduced the antimalarial activity. Alkyl substituents in the para positions (**4b**) confer the best activity against CQ-sensitive 3D7 and CQ-resistant K1 strains (Figure 6).

## 3. Materials and Methods

### 3.1. General Methods

All chemicals and solvents (methanol, ethanol, toluene, dimethyl sulfoxide) were obtained from commercially available sources and used without further purification. 4,7-Dichloroquine, 97% hydrobromic acid 48%, monoethanolamine, activated charcoal (granulated), diethyl oxalacetate sodium salt 95%, benzaldehyde 98%, hydrazine solution 35 wt.% in H_2_O, 4-ethylbenzaldehyde 98%, and isobutyraldehyde 99% were purchased from Sigma-Aldrich, Darmstadt, Germany. Furaldehyde was supplied by Fischer Chemical (Pittsburgh, PA, USA), while 4-hydroxybenzaldehyde was 99% (ACROS Organics, Geel, Belgium). ^1^H and ^13^C NMR spectra were recorded on a Joel Resonance ECZ400S 400 MHz spectrometer (Selangor, Malaysia) at 400 MHz and 100 MHz, respectively, using TMS as an internal standard in CDCl_3_ *d_6_*-dimethyl sulphoxide (DMSO) solutions. Elemental analyses were performed on a Flash Elemental Analyzer (Selangor, Malaysia) 110 series. The melting point was determined on the melting point apparatus, Stuart SMP40, at room temperature until 400 °C. The progress of the reactions was monitored by thin-layer chromatography (TLC) on silica gel from Merck Kieselgel (New Jersey, NJ, USA) 60F_254,_ and the product was visualized with an ultraviolet lamp.

### 3.2. General Procedure for the Synthesis of Pyrano[2,3-c]pyrazole

#### 3.2.1. Synthesis of Pyrano[2,3-c]pyrazole-3-carboxylate (**1a–e**)

Synthesis of pyrano[2,3-c]pyrazole-3-carboxylate **1a–e** compounds has been reported by Mohammat et al. via a four-component reaction. The spectral and analytical data were in agreement with previously published data [30].

#### 3.2.2. Synthesis of 4-(Ethanolamino)-7-chloroquine (**2**)

A mixture of 4,7-dichloroquinoline (0.99 g, 5 mmol) and ethanolamine (1.5 mL, 25 mmol) was mixed in a three-neck round-bottom flask. The reaction was refluxed for 180 min, and the temperature was monitored using a thermometer. The reaction flask was cooled with an ice-water bath and stirring was continued until the precipitation of a colorless solid. Yield: 1.04 g (93%); Melting point: 210–211 °C; ^1^H NMR (400 MHz, DMSO): δ 8.34 (d, *J* = 5.0 Hz, 1H), 8.21 (d, *J* = 8.7 Hz, 1H), 7.74 (d, *J* = 2.3 Hz, 1H), 7.40 (dd, *J* = 9.1, 2.3 Hz, 1H), 7.24 (t, *J* = 5.5 Hz, 1H), 6.46 (d, *J* = 5.5 Hz, 1H), 4.81 (t, *J* = 5.5 Hz, 1H), 3.62 (q, *J* = 5.8 Hz, 2H), 3.30 (q, *J* = 5.8 Hz, 2H); ^13^C NMR (100 MHz, DMSO) δ 45.6 (CH_2_N), 59.3 (CH_2_Br), 99.2 (Ar C), 118.0 (quat. C), 124.6 (Ar C), 124.6 (Ar C), 128.0 (Ar C), 133.9 (quat. C), 149.6 (quat. C), 150.8 (quat. C), 152.4 (Ar C).

#### 3.2.3. Synthesis of 4-(Bromoethylamino)-7-chloroquinoline (**3**)

To a solution of hydrogen bromide, 33% (5 mL, 30 mmol), 4-(ethanolamine)-7-chloroquinoline (1.1135 g, 5 mmol) was added dropwise, together with sulfuric acid (0.5 mL, 10 mmol). The resulting mixture was refluxed for 60 min, and the progress of the reaction was monitored using TLC. The reaction mixture was left to cool at room temperature and poured into ice water. The solution was added with ammonia hydroxide until pH 9 to yield a solid colorless precipitate. The solid was filtered and heated in a solution of toluene and activated charcoal. The mixture was filtered and refrigerated at 4 °C to yield a colorless solid. Yield: 0.57 g (40%); Melting point: 140–141 °C; ^1^H NMR (400 MHz, DMSO): δ 8.36 (d, *J* = 5.5 Hz, 1H), 8.06 (d, *J* = 9.1 Hz, 1H), 7.77 (d, *J* = 2.3 Hz, 1H), 7.40 (dd, *J* = 8.7, 2.3 Hz, 1H), 6.56 (d, *J* = 5.5 Hz, 1H), 3.79 (t, *J* = 6.6 Hz, 2H), 3.64 (t, *J* = 6.4 Hz, 2H); ^13^C NMR (100 MHz, CD_3_OD): δ 28.9 (CH_2_N), 44.3 (CH_2_Br), 98.4 (ArC), 117.4 (quat. C), 122.9 (ArC), 125.0 (ArC), 126.3 (ArC), 135.2 (quat. C), 148.3 (quat. C), 150.9 (quat. C), 151.1 (ArC).

### 3.3. General Procedure for the Synthesis of Hybrid *(****4a*–*e****)*

**Figure 7:**
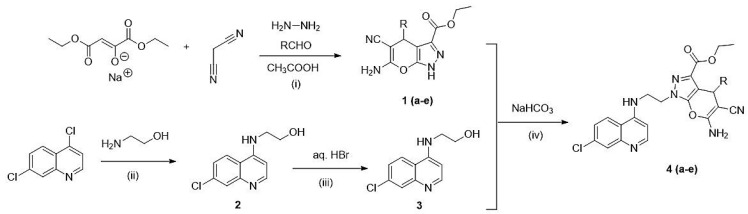
Reagents and conditions: (i) EtOH, reflux 1–2 h, *R* = benzaldehyde, 4-ethylbenzaldehyde, isobutyraldehyde, 4-hydroxybenzaldehyde and furaldehyde; (ii) reflux 1h; (iii) reflux 3 h and (iv) DMSO, r.t.

A mixture of 4-(bromoethylamino)-7-chloroquinoline (1.4284 g, 5 mmol) and corresponding pyrano[2,3-c]pyrazole-3-carboxylate **1a–e** compounds (1.5515 g, 5 mmol) and sodium bicarbonate (0.8401 g, 10 mmol) were mixed in a DMSO at 30–40 °C for 24 h. The mixture was left to cool at room temperature and extracted using a mixture of 20 mL ethyl acetate and 80 mL of brine. The organic layer was separated, concentrated using a rotary evaporator, and purified using silica column chromatography by elution with a mixture of ethanol:ethylacetate: hexane (1:3:1) (Figure 7).

#### 3.3.1. Ethyl 6-Amino-1-(2-((7-chloroquinolin-4-yl)amino)ethyl)-5-cyano-4-phenyl-1,4-dihydropyrano[2,3-c]pyrazole-3-carboxylate (**4a**)

Yield: 0.34 g (13%); Melting point: 260–261 °C; ^1^H NMR (400 MHz, DMSO):δ 8.33 (d, *J* = 5.0 Hz, 1H), 8.00 (d, *J* = 9.1 Hz, 1H), 7.74 (d, *J* = 2.3 Hz, 1H), 7.35 (dd, *J* = 8.9, 2.1 Hz, 1H), 7.35(m, 1H), 7.21 (t, *J* = 7.3 Hz, 2H), 7.14 (t, *J* = 7.3 Hz, 1H), 6.98 (d, *J* = 6.8 Hz, 2H), 6.98 (s, 2H), 6.43 (d, *J* = 5.5 Hz, 1H), 4.75–4.41 (m, 2H), 4.6 (s, 1H) 3.74–3.51 (m, 2H), 3.74–3.51 (m, 2H), 0.68 (t, *J* = 7.1 Hz, 3H); ^13^C NMR (100 MHz, DMSO): δ 160.2 (CNH_2_), 158.6 (C=O), 154.8 (quat. Ar C), 152.3 (Ar C), 150.2 (quat. Ar C), 149.6 (quat. Ar C) 145.3 (quat. Ar C), 133.9 (quat. Ar C), 129.6 (quat. Ar C), 128.8 (Ar C), 128.0 (Ar C), 127.6 (Ar C), 127.1 (Ar C), 124.7 (Ar C), 124.3 (Ar C), 120.7 (CN), 117.9 (quat. C), 105.5 (quat. C), 99.1 (Ar C), 61.3 (CH_2_C), 58.5 (quat. C), 49.7 (CH_2_N), 42.7 (CH_2_N), 37.8 (CH), 13.7 (CH_3_). Anal. Calc. for C_27_H_23_ClN_6_O_3_, C 62.97, H 4.50, N 16.32. Found: C 62.42, H 4.47, N 16.29.

#### 3.3.2. Ethyl 6-Amino-1-(2-((7-chloroquinolin-4-yl)amino)ethyl)-5-cyano-4-(4-ethylphenyl)-1,4-dihydropyrano[2,3-c]pyrazole-3-carboxylate (**4b**)

Yield: 0.41 g (15%); Melting point: 145–147 °C; ^1^H NMR (400 MHz, DMSO): δ 8.32 (d, *J* = 5.5 Hz, 1H), 7.98 (d, *J* = 9.1 Hz, 1H), 7.73 (d, *J* = 2.4 Hz, 1H), 7.34 (dd, *J* = 8.7, 2.3 Hz, 1H), 7.34 (m, 1H), 7.02 (d, *J* = 7.8 Hz, 2H), 6.95 (s, 2H), 6.85 (d, *J* = 8.2 Hz, 2H), 6.42 (d, *J* = 5.5 Hz, 1H), 4.61 (dt, *J* = 10, 4.4 Hz, 1H), 4.57 (s, 1H), 4.08 (q, *J* = 5.2 Hz, 2H), 3.69–3.60 (m, 2H), 3.59 (m, 2H), 2.51 (t, *J* = 7.5 Hz, 2H), 1.09 (t, *J* = 7.5 Hz, 3H), 0.63 (t, *J* = 7.1 Hz, 3H). ^13^C NMR (100 MHz, DMSO): δ 160.1 (CNH_2_), 158.6 (C=O), 154.8 (quat. Ar C), 152.3 (Ar C), 150.2 (quat. Ar C) 149.5 (quat. Ar C), 142.6 (quat. Ar C),142.4 (quat. Ar C), 133.9 (quat. Ar C), 129.7 (quat. Ar C), 128.1 (Ar C), 127.9 (Ar C), 127.5 (Ar C), 124.7 (Ar C), 117.9 (Ar C), 120.8 (CN), 117.9 (CNH), 105.6 (quat. C), 99.1 (Ar C), 61.2 (CH_2_C), 58.7 (quat. C), 49.7 (CH_2_N), 42.4 (CH_2_N), 37.4 (CH), 28.3 (CH_2_C), 16.1 (CH_3_), 13.8 (CH_3_). Anal. Calc. for C_29_H_27_ClN_6_O_3_, C 64.14, H 5.01, N 15.48. Found: C 62.99, H 5.02, N 15.38.

#### 3.3.3. Ethyl 6-Amino-1-(2-((7-chloroquinolyn-4-yl)amino)ethyl)-5-cyano-4-(furan-2-yl)-1,4-dihydropyrano[2,3-c]pyrazole-3-carboxylate (**4c**)

Yield: 0.51 g (20%); Melting point: 232–234 °C; ^1^H NMR (400 MHz, DMSO): δ 8.34 (d, *J* = 5.5 Hz, 1H), 8.03 (d, *J* = 9.1 Hz, 1H), 7.75 (d, *J* = 2.3 Hz, 1H), 7.43 (d, *J* = 1.4 Hz, 1H), 7.40 (dd, *J* = 9.1, 2.3 Hz, 1H), 7.36 (t, *J* = 6.2 Hz, 1H), 7.10 (s, 2H), 6.44 (d, *J* = 5.5 Hz, 1H), 6.28 (t, *J* = 1.7 Hz, 1H), 5.97 (d, *J* = 3.2 Hz, 1H), 4.79 (s, 1H), 4.76–4.44 (m, 2H), 3.85–3.69 (m, 2H), 3.64 (t, *J* = 5.7 Hz, 2H), 0.86 (t, *J* = 7.1 Hz, 3H).). ^13^C NMR (100 MHz, DMSO): δ 161.1 (CNH_2_), 158.7 (C=O), 156.1 (quat. C), 154.6 (quat. Ar C), 152.3 (Ar C), 150.1 (quat. Ar C) 149.6 (quat. Ar C), 133.9 (quat. Ar C), 129.8 (quat. C), 128.0 (Ar C), 124.8 (Ar C), 124.3 (Ar C), 120.6 (CN), 117.9 (CNH), 110.8 (Ar C), 105.7 (Ar C), 103.0 (quat. C), 99.1 (Ar C), 61.5 (CH_2_C), 55.3 (quat. C), 49.7 (CH_2_N), 42.7 (CH_2_N), 31.6 (CH), 13.9 (CH_3_). Anal. Calc. for C_25_H_21_ClN_6_O_4_, C 59.47, H 4.19, N 16.64. Found: C 59.15, H 4.12, N 16.64.

#### 3.3.4. Ethyl 6-Amino-1-(2-((7-chloroquinolyn-4-yl)amino)ethyl)-5-cyano-4-isopropyl-1,4-dihydropyrano[2,3-c]pyrazole-3-carboxylate (**4d**)

Yield: 0.24 g (10%); Melting point: 221–223 °C; ^1^H NMR (400 MHz, DMSO): δ 8.31 (d, *J* = 5.5 Hz, 1H), 8.01 (d, *J* = 9.1 Hz, 1H), 7.74 (d, *J* = 11.7 Hz, 1H), 7.38 (dd, *J* = 9.2, 2.0 Hz, 1H), 7.36 (t, *J* = 6.0 Hz, 1H), 7.01 (s, 2H), 6.41 (d, *J* = 5.5 Hz, 1H), 4.76–4.48 (m, 2H), 4.00–3.80 (qd, *J* = 7.6, 3.6 Hz, 2H) 3.63 (t, *J* = 5.9 Hz, 2H), 3.47 (d, *J* = 2.7 Hz, 1H), 1.89–1.69 (qqd, *J* = 6.8, 2.8 Hz, 1H), 1.04 (t, *J* = 7.1 Hz, 3H), 0.87 (d, *J* = 7.3 Hz, 3H), 0.51 (d, *J* = 6.9 Hz, 3H). ^13^C NMR (100 MHz, DMSO): δ 163.4 (CNH_2_), 159.2 (C=O), 156.5 (quat. C), 152.3 (Ar C), 150.2 (quat. Ar C) 149.5 (quat. Ar C), 134.0 (quat. Ar C), 128.7 (quat. C), 127.9 (Ar C), 124.7 (Ar C), 124.3 (Ar C), 122.2 (CN), 117.9 (CNH), 106.9 (Ar C), 99.1 (Ar C), 61.6 (CH_2_C), 51.8 (quat. C), 49.8 (CH_2_N), 42.7 (CH_2_N), 38.0 (CH), 35.7 (CH), 20.6 (CH_3_), 17.1 (CH_3_), 13.9 (CH_3_). Anal. Calc. for C_24_H_25_ClN_6_O_3_, C 59.94, H 5.24, N 17.47. Found: C 59.76, H 5.19, N 17.66.

#### 3.3.5. Ethyl 6-Amino-1-(2-((7-chloroquinolyn-4-yl)amino)ethyl)-5-cyano-4-(4-hydroxyphenyl)-1,4-dihydropyrano[2,3-c]pyrazole-3-carboxylate (**4e**)

Yield: 0.13 g (5%); Melting point: 233–234 °C; ^1^H NMR (400 MHz, DMSO): δ 9.25 (s, 1H), 8.33 (d, *J* = 5.5 Hz, 1H), 8.00 (d, *J* = 9.1 Hz, 1H), 7.74 (d, *J* = 2.3 Hz, 1H), 7.36 (dd, *J* = 8.7, 2.3 Hz, 1H), 7.36 (m, 1H) 6.94 (s, 2H), 6.77 (d, *J* = 8.7 Hz, 2H), 6.59 (d, *J* = 8.7 Hz, 2H), 6.42 (d, *J* = 5.5 Hz, 1H), 4.70–4.51 (m, 2H), 4.51 (s, 1H), 3.74–3.51 (m, 2H), 3.74–3.51 (m, 2H), 0.74 (t, *J* = 7.1 Hz, 3H). ^13^C NMR (100 MHz, DMSO): δ 160.0 (CNH_2_), 158.7 (C=O), 156.5 (quat. C), 154.7 (quat. Ar C), 152.3 (Ar C), 150.1 (quat. Ar C) 149.6 (quat. Ar C), 135.8 (quat. Ar C), 133.9 (quat. C), 129.6 (quat. C), 128.6 (Ar C), 128.0 (Ar C), 124.7 (Ar C), 124.4 (Ar C), 120.8 (CN), 117.9 (CNH), 115.4 (Ar C), 106.2 (quat. C), 99.1 (Ar C), 61.3 (CH_2_C), 59.0 (quat. C), 49.7 (CH_2_N), 42.7 (CH_2_N), 37.0 (CH), 13.8 (CH_3_). Anal. Calc. for C_27_H_23_ClN_6_O_4_, C 61.08, H 4.37, N 15.83. Found: C 60.97, H 4.72, N 15.95.

### 3.4. Antimalarial Assessments of Hybrid Molecules *(****4a*–*e****)* Using In Vitro P. falciparum 3D7 and K1 Cultures

#### 3.4.1. Antimalarial Assay

The parasites were cultivated by the Trager and Jensen methods [31] to determine the antimalarial activity of the hybrid 4-aminoquinoline-pyrano[2,3-c]pyrazole derivatives against chloroquine-sensitive *P. falciparum* 3D7 and chloroquine-resistant *P. falciparum* K1. *P. falciparum* 3D7 (chloroquine-sensitive) (MRA-102), and *P. falciparum* K1 (chloroquine-resistant) (MRA-159) were originally obtained from BEI. Resources, NIAID, NIH (*P. falciparum*, Strain 3D7, MRA-102, contributed by Daniel J. Carucci; *P. falciparum*, Strain K1, MRA-159, contributed by Dennis E. Kyle). The parasites were maintained in continuous culture in fresh group O (Rhesus positive) human erythrocytes suspended in 2% hematocrit in RPMI 1640 containing 10% Albumax I, glucose (3 g/L), hypoxanthine (45 μg/L), and gentamicin (50 μg/L). Plasmodium lactate dehydrogenase (pLDH) assays were performed as described by Makler et al., with slight modification [32,33]. Parasites were plated in asynchronized phase at 2% hematocrit and 2% parasitemia in 100 μL of the test compounds. The compounds were dissolved in DMSO and further diluted in a culture medium. Chloroquine diphosphate and artemisinin were used as antimalarial reference drugs. Unparasitized erythrocytes without the test compounds were the blanks, while parasitized erythrocytes without test compounds were the control for the assay. Plates were placed in an incubator supplied with 5% CO_2_ and incubated at 37 °C for 48 h. After incubation, plates were subjected to three 30 min of freeze-thaw cycles to lyse and re-suspend the erythrocytes in the culture. Subsequently, Malstat reagent (100 μL) and a nitro blue tetrazolium salt/phenazine ethosulfate (NBT/PES) solution (25 μL) were added to each well of a new 96-well microtiter plate. The culture (25 μL) was then added to the corresponding well of the reagent plate to initiate the lactate dehydrogenase reaction. Color development of the plate was monitored calorimetrically at 650 nm after an hour of incubation in the dark. Data obtained was analyzed through non-linear regression via Graph pad prism software to obtain EC_50_ values (inhibition concentration at 50% parasite growth).

#### 3.4.2. Cytotoxicity Assay

The cytotoxicity of the hybrid 4-aminoquinoline-pyrano[2,3-c]pyrazole derivatives was measured using the MTT assay described by Mossmann [34]. Before performing the assay, 100 μL of Vero cells (ATCC CCL-81) in culture medium supplemented with 10% fetal bovine serum (FBS) were seeded in a 96-well flat-bottom microtiter plate at 2 × 10^4^ cells/well and allowed to adhere for 48 h at 37 °C in a CO_2_ incubator. After 48 h of incubation, the confluent cells were treated with the test compounds (10 to 0.01 μg/mL) to each well accordingly. The cell suspension without test compounds served as the positive control for cell growth. After 48 h incubation, MTT reagent in phosphate buffer saline (5 mg/mL) was added to each well, followed by 3 h incubation at 37 °C. The medium was then aspirated, and formazan crystals were solubilized by adding 100 μL of DMSO per well for 10 min at 37 °C in a CO_2_ incubator. The solution was measured at 540 nm with a microplate reader. Three independent tests were conducted to determine the cytotoxicity of each test compound. The percentage of growth inhibition and the 50% cytotoxic concentration (CC_50_) was estimated from a non-linear regression using Graphpad Prism. Cytotoxicity was expressed as the concentration of the test compounds inhibiting cell growth by 50% (CC_50_).

#### 3.4.3. Selectivity and Resistance Indexes Calculation

Selectivity indexes of the hybrid 4-aminoquinoline-pyrano[2,3-c]pyrazole derivatives were determined by a ratio of values from CC_50_ of cytotoxicity and EC_50_ of antimalarial activities (MTT/pLDH). Test pure compounds with SI values greater than 100 (SI > 100) are considered strong and selective antimalarial agents [35]. Resistance index is the ratio of the EC_50_ for the resistant versus the sensitive strain (K1/3D7). The RI is a quantitative measurement of the antimalarial activity against the chloroquine-resistance strain relative to that against the chloroquine-sensitive strain. A lower RI index value indicates higher potency of a compound against chloroquine-resistant malarial parasites [36].

### 3.5. Computational Studies

#### 3.5.1. DFT Calculations

All compounds were modeled (GaussView 5.0) and optimized using density functional theory (DFT) with the B3LYP/6–31++G theoretical model and the Gaussian09 program [37]. The vibrational frequency calculations were established and validated to ensure that only positive eigenvalues were obtained.

#### 3.5.2. Preparation and Optimization of Ligands

A series of hybrid compounds were constructed using Marvin Sketch version 1.5.6. These ligand structures were converted into 3D formats and geometrically optimized with OPLS3 using Ligand Preparation Wizard in Schrodinger Small Molecule Drug Discovery Suite 2017-1.

#### 3.5.3. Preparation and Optimization of Protein Receptors and Molecular Docking

The X-ray crystal structures of the protein-ligand complex for PfLDH (PDB IDs: 1CET and 1U5A) were retrieved from the Protein Database Bank (PDB) (http://www.rcsb.org, accessed on 12 May 2020). All water molecules of the crystal structures were removed, while hydrogen atoms were added in their standard geometry, adjusting the bond orders and formal charges. The ligand-binding site was identified based on the position of the inhibitor-binding site, as reported in the literature. The in-house CDOCKER protocol was used for the docking analysis of active compounds to the binding site. Different conformations were performed for each ligand through high-temperature molecular dynamics. The ligands were heated (700 K in 2000 steps) followed by cooling (300 K), and they were then subjected to refinement by grid-based (GRID 1) simulated annealing and full force minimization after random rotation. The ligand was allowed to flex while the receptor was held rigid during the refinement process. The generated ligand conformations were clustered according to their binding interactions. For validation of the docking protocol, the co-crystallized ligands were re-docked to the binding site.

#### 3.5.4. Pharmacological Properties of the Hybrid Compounds

The pharmacological properties of the synthesized hybrid compounds were measured using the standard ADMET descriptors protocol implemented in Discovery Studio 3.1. The parameters involved in the analysis were plasma–protein binding (PPB), human intestinal absorption (HIA), atom-based log P (Alog P98), aqueous solubility, polar surface area (PSA), blood–brain barrier (BBB) penetration, cytochrome P450 2D6 (CYP2D6) enzyme inhibition, and hepatotoxicity.

## 4. Conclusions

In summary, we developed a simple reaction protocol for a new series of antimalarial hybrid compounds containing pyrano[2,3-c]pyrazole-aminoquinoline pharmacophoric units. From the antimalarial assessment, compound **4b** is considered the most potent, with high a selectivity index against both the 3D7 and K1 strains and a low resistance index value. The compound also exerted high-affinity binding on PfLDH, which suggests a potential molecular target in malarial infection. The findings from pharmacophoric hybridization in this study are crucial as an alternative therapy to combat drug resistance in malarial infection. Further, in vivo, antimalarial activity needs to be carried out, and we envisage the expansion of a hybrid antimalarial medicine series based on these derivatives.

## Figures and Tables

**Table 1:** Antimalarial and cytotoxic activities of hybrid 4-aminoquinoline-pyrano[2,3-c]pyrazole derivatives.

Test Compounds	*P. falciparum* 3D7 (CQ-Sensitive) EC_50_ (µM) ± S.D.	*P. falciparum* K1 (CQ-Resistant) EC_50_ (µM) ± S.D.	Vero Cell (Normal Cell) CC_50_ (µM) ± S.D.	Selectivity Index (SI), $\left(\frac{CC_{50}\;\mathrm{MTT}}{{\mathrm{EC}}_{50}\;\mathrm{pLDH}}\right)$		Resistance Index (RI), $\left(\frac{{\mathrm{EC}}_{50}\;\mathrm{Resistance}}{{\mathrm{EC}}_{50}\;\mathrm{Sensitive}}\right)$
				CQ-Sensitive	CQ-Resistant	
**4a**	0.19 ± 0.07	0.25 ± 0.03	102.54 ± 22.15	539.7	410.16	1.32
**4b**	0.0130 ± 0.0002	0.02 ± 0.01	17.60 ± 1.50	1353.9	880	1.53
**4c**	0.113 ± 0.002	1.61 ± 0.15	84.52 ± 6.45	747.9	52.49	14.24
**4d**	3.39 ± 1.89	0.30 ± 0.01	86.14 ± 7.33	25.4	287	0.09
**4e**	0.026 ± 0.009	7.12 ± 3.72	9.24 ± 1.13	355.3	1.3	273.84
4-aminoquinoline	9.29 ± 0.12	140.43 ± 27.61	113.85 ± 1.63	12.25	0.81	15.11
CQ	0.002	0.33	138.40 ± 8.77	>2000	>2000	16.5
ART	0.0001	0.00017	434.60 ± 64.21	>2000	>2000	1.7

S.D., standard deviation; EC_50_, half-maximal effective concentration; CC_50_, half-maximal cytotoxic concentration; CQ, chloroquine; ART, artemisinin.

**Table 2:** Docking results for compounds **4a**–**e** and 3,5-dihydroxy-2-naphthoic acid in the active site of PfLDH.

Compounds	Cdocker Energy (kcal/mol)	Cdocker Interaction Energy (kcal/mol)	Residues of the Active Site Interacting with the Ligands
**4a**	−13.7	−53.9	Thr97, Thr101, Trp102, Ser245, Ser245, Pro246, Val240, Asn102, Leu237, Glu102, Pro141, Asn234, Asn197, Gly196, Val233, Met199, Asp168, Arg171, His195, Asn140, Leu167, Tyr247, Pro250, Ile31, Val138, Thr139, Thr97
**4b**	−20.7 (* −16.4)	−60.5 (* −54.3)	Refer to Figure 5d * (Figure 5f)
**4c**	−19.4	−56.9	Ile31, Thr97, Val138, Thr139, Pro250, Arg171, Leu167, His195, Pro141, Val142, Asn140, Gly196, Asn197, Asb234, Val233, Met199, Val240, Asn102, Leu237, Ala236, Pro246, Trp102, Ser245, Thr101
**4d**	−19.0	−53.7	Gly29, Met30, Thr97, Thr101, Gly99, Phe100, Thr139, Lys102, Glu102, Asn140, Pro141, Val142, His195, His196, Val233, Asn197, Asn234, Met199, Leu237, Ala236, Asn102, Val240, Trp102, Pro246, Tyr247, Ile31
**4e**	−17.4	−56.9	Met30, Ile31, Thr97, Thr101, Thr139, Val138, Leu163, Pro246, Leu167, Ala236, Asn140, Leu167, His195, Asn197, Met199, Arg171, Gly196, Val233, Asp168, Glu102, Asn102, Leu237, Trp102, Val240, Ser245, Tyr247
3,5-dihydroxy-2-naphthoic acid	−32.7	−44.5	Gly196, Asn102, Asn197, Trp102, Ala236, Val240, Pro246, Pro250, Leu167, Asn140, Arg171, His195

* the best binding pose in the second cluster result. For the color representation, black, van der Waals interaction, etc.; green, hydrogen bonding interaction; purple, ᴨ-σ, ᴨ-ᴨ interaction; brown, salt bridge, attractive charge interaction.

**Table 3:** ADMET profile prediction of compounds **4a**–**e**.

Compound	ADMET Parameter									
	Human Intestinal Absorption			Aqueous Solubility		Blood-Brain Barrier (BBB) Penetration		Plasma Protein Binding (PPB)	Cytochrome P_450_ 2D6 (CYP2D6)	Hepatotoxicity
	PSA ^a^	ALogP98 ^b^	Level ^c^	Log(Sw) ^d^	Level ^e^	LogBB ^f^	Level ^g^	Prediction ^h^	Prediction ^i^	Prediction ^j^
**4a**	125.316	4.577	2	−6.68	1	-	4	0	0	1
**4b**	125.316	5.519	2	−7.285	1	-	4	0	0	1
**4c**	146.132	4.335	2	−6.601	1	-	4	0	0	1
**4d**	137.870	3.761	2	−6.278	1	-	4	0	0	1
**4e**	125.316	4.252	2	−6.402	1	-	4	0	0	1

^a^ Polar surface area (PSA) (>150: very low absorption); ^b^ Atom-based log P (Alog P98) (≤−2.0 or ≥7: very low absorption); ^c^ Level of human intestinal absorption prediction; 0 (good), 1 (moderate), 2 (poor), 3 (very poor); ^d^ The based ^10^logarithm of the molar solubility log (Sw) (25 °C, pH = 7.0) (acceptable drug-like compounds: −6 < log(Sw) ≤ 0); ^e^ Level of aqueous solubility prediction; 0 (extremely low), 1 (very low), 2 (low), 3 (good), 4 (optimal), 5 (too soluble), 6 (warning: molecules with one or more unknown Alog P calculation); ^f^ Very high penetrants (log BBP ≥7); ^g^ Level blood brain barrier penetration prediction; 0 (very high penetrate), 1 (high), 2 (medium), 3 (low), 4 (undefined); ^h^ Prediction plasma-protein binding (0: <90%; 1: ≥90%;); ^i^ Prediction cytochrome P_450_ 2D6 enzyme inhibition (0, non-inhibitor; 1, inhibitor); ^j^ Prediction hepatotoxicity (0, non-toxic; 1, toxic).

## Data Availability

The data presented in this study are available in this article.
